# A look back at 3 years of neurological research and practice (NRP)

**DOI:** 10.1186/s42466-022-00182-z

**Published:** 2022-04-28

**Authors:** Werner Hacke

**Affiliations:** University Hospital Heidelberg, Rubrechts-Karl-University Heidelberg, Im Neuenheimer Feld 400, 69120 Heidelberg, Germany

In March 2019, we launched Neurological Research and Practice (NRP), the official English language, open access, online journal of the German Society of Neurology (Deutsche Gesellschaft für Neurologie) [1]. Each year, I have reported on the new journal's progress [2, 3]. In our third year, it’s time again to summarize what we've achieved and what's about to come.

I'm proud to say that we have achieved several significant milestones. First, NRP has now published more than 150 peer-reviewed articles. As submissions have increased, our acceptance rate has dropped from around 60% to below 40%. While most articles originate from Germany, about 30% have first authors and affiliations from outside the country. This number has also increased over the years.

**Fig. 1:**
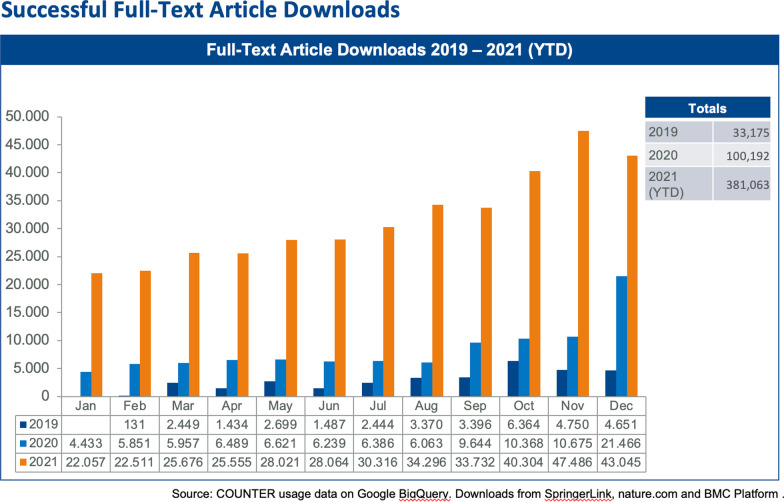
Successful full-article downloads

**Fig. 2:**
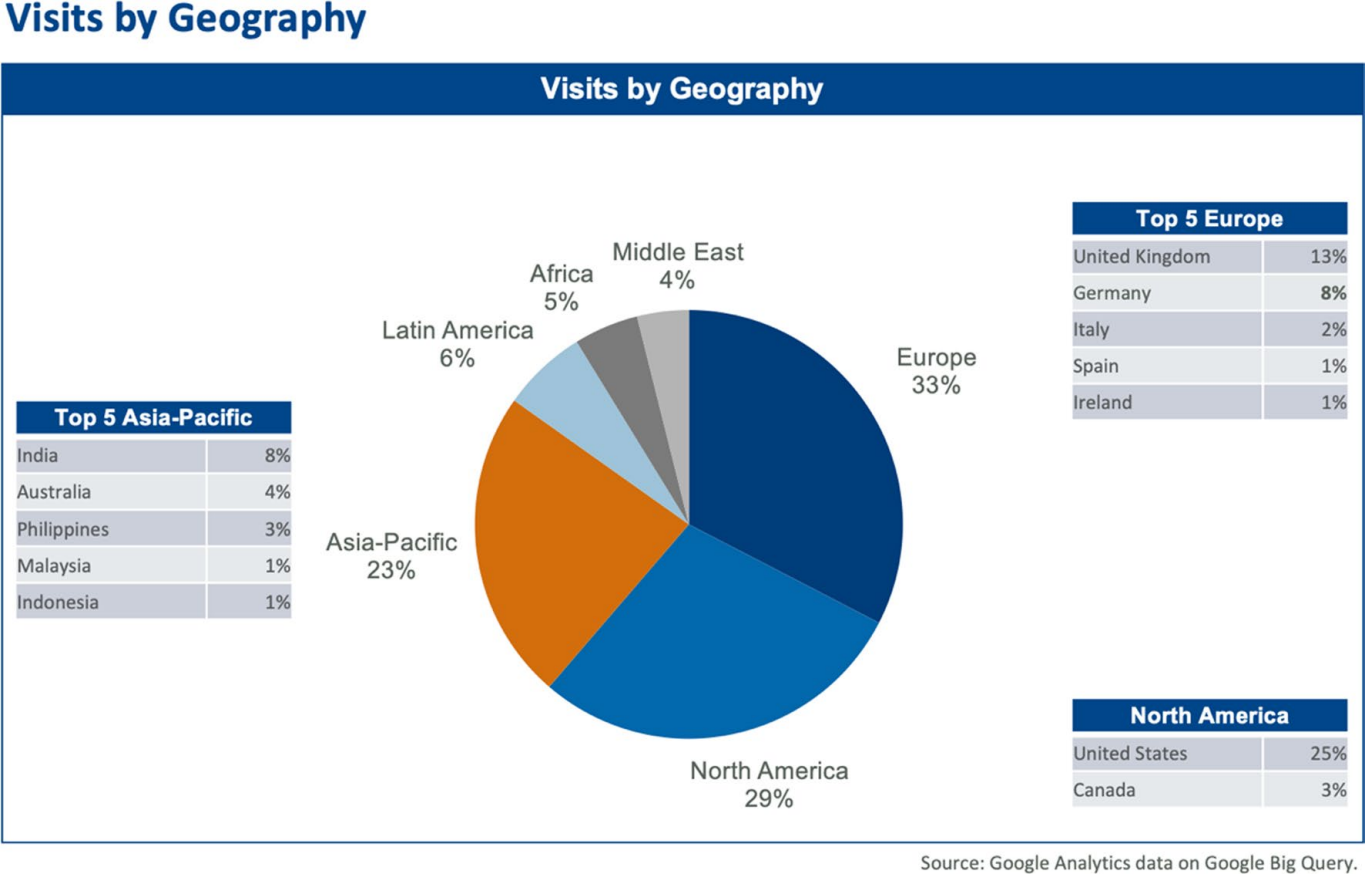
Visits by geography

Second, last year NRP was listed in PubMed®, a major accomplishment for a journal less than three years after its first publication. PubMed® was largely responsible for the remarkable increase in full article downloads (Fig. 1) and international visits to the journal's website (Fig. 2). These two graphs show how access to our articles has continuously grown and from which global regions they are being made. The international visits are impressive. Surprisingly, the number of hits from Germany is relatively low compared with the numbers from the U.S., Great Britain, and India.

Third, the citation of articles is still one of the most prominent aspects of the impact and visibility of a journal. For NRP, the citation rates are quite good, especially for a new journal. Table 1 lists the most cited articles from 2019 and 2020 (with reference numbers). The most frequently cited were not only research articles and reviews but also guidelines, SOPs, letters, and a clinical trial protocol indicating that readers found valuable information in a wide range of our publication categories. Another important feature is the number of article accesses. Table 2 lists articles with more than 7000 accesses. The most accessed article already has more than 150,000 accesses (there's some overlap with the most cited articles, but Table 2 includes more articles from 2021, and the most accessed articles were not necessarily the most cited ones).

**Table 1:** Highly cited articles (> 10 citations)

	References	Year	Citations	Type
Dziewas et al.	[4]	2019	41	res
Berlit et al.	[5]	2020	35	gui
Grefkes and Fink	[6]	2019	30	rev
Weber et al.	[7]	2019	30	res
Mokli et al.	[8]	2019	28	rev
Busetto et al.	[9]	2020	28	rev
Lehmann et al.	[10]	2020	20	sop
Lampe et al.	[11]	2020	17	let
Prasuhn et al.	[13]	2019	14	tri
Sembill et al.	[12]	2019	13	rev
Göttle et al.	[14]	2019	12	rev
Schlereth	[15]	2020	12	gui
Berg et al.	[16]	2020	12	gui
Eyding et al.	[17]	2019	11	res
Marsh	[19]	2019	11	rev

gui, guideline; let, letter; res, research article; rev, review; sop, standard operating procedure; tri, trial protocol

**Table 2:** Top 20 accesses > 7000

	References	Year	Accesses	
Busetto et al.	[9]	2020	169.000	rev
Berlit et al.	[5]	2020	34.000	gui
Werner et al.	[20]	2020	24.000	let
Grefkes et al.	[6]	2020	19.000	rev
Lehmann et al.	[10]	2020	19.000	sop
Sembill et al.	[12]	2019	15.000	rev
Gövert et al.	[21]	2020	14.000	rev
Mokli et al.	[8]	2019	9.500	rev
Bien and Bien	[22]	2020	8.600	rev
Schlereth et al.	[15]	2020	8.500	gui
Stetefeld and Schroeter	[23]	2019	8.000	sop
Grobe-Einsler and Kaut	[24]	2020	8.000	let
Ganti et al.	[25]	2019	7.600	res
Meyding-Lamade´ et al.	[26]	2019	7.400	rev
Walter and Kremer	[27]	2021	7.200	let
Stefanou et al.	[28]	2019	7.100	rev
Rössling and Prüss	[29]	2020	7.000	sop

gui, guideline; let, letter; res, research article; rev, review; sop, standard operating procedure; tri, trial protocol

One of the next milestones we are looking forward to is NRP becoming indexed in the Emerging Sources Citation Index produced by Clarivate Analytics. Becoming indexed would further improve the journal's visibility and contribute to its Impact Factor. After just three years in operation, this would be a major achievement. Let's keep our fingers crossed that I will be able to report on that next year.

At the beginning of 2022, after 3 years of me being the sole editor of the Journal, we appointed section editors for different scientific areas (you can find a list of section editors on the website starting page. https://neurolrespract.biomedcentral.com). The section editors, international editorial board members, and many other colleagues have supported NRP as authors and reviewers. Our single, blinded peer review protocol requires at least two and sometimes more reviews. As we all know, it's getting increasingly difficult to find qualified reviewers who commit to the swift delivery of high-quality reviews, especially for a new journal. Unfortunately, Data Protection Regulations prevent us from listing the reviewers who supported the Journal and express our gratefulness for their work. Some of the quick and reliable reviewers have been invited frequently and contributed a lot. The list is led by 5 colleagues, who contributed more than 15 Reviews. These are Christos Krogias [22] from Bochum, Simon Nagel from Heidelberg, now Ludwigshafen [16]. Uta Meyding-Lamade´ from Frankfurt [20], Ralf Linker from Regensburg [17], and Julian Bösel from Kassel [15]. These colleagues have agreed to the publication of their names. In order to acknowledge the contributions of our reviewers we will create a regularly updated Top reviewers list on the journals webpage for further reference. Unfortunately, we also had some colleagues on the editorial board and in our growing list of potential reviewers who never responded, declined every invitation, or did not deliver although they initially agreed to review. However, this is not specific to our journal, and I hear that from several fellow editors of other international journals. Spam filters and hospital firewalls may be partially responsible for that.

All in all, we have had a very rewarding first 3 years of the journal. Once we receive an Impact Factor, we can declare that NRP is out of "early childhood" and about to enter the next phase of its development.

Thank you to all who supported this endeavor and helped with a successful takeoff.
